# Long-Term Grazing Exclusion Reveals Taxonomic and Functional Reorganization of Plant Communities in an Insular Mediterranean Geopark

**DOI:** 10.3390/plants15111692

**Published:** 2026-05-30

**Authors:** Vasiliki Kakampoura, Yiannis G. Zevgolis, Nikolaos Zouros, Maria Panitsa, Panayiotis G. Dimitrakopoulos

**Affiliations:** 1 Biodiversity Conservation Laboratory, Department of Environment, University of the Aegean, 81132 Mytilene, Greece; vkakampoyra@gmail.com (V.K.); zevgolis@env.aegean.gr (Y.G.Z.); 2 Department of Geography, University of the Aegean, 81132 Mytilene, Greece; nzour@aegean.gr; 3 Laboratory of Botany, Department of Biology, Division of Plant Biology, University of Patras, 26504 Patras, Greece; mpanitsa@upatras.gr

**Keywords:** grazing exclusion, plant community assembly, functional traits, biodiversity patterns, UNESCO Global Geopark

## Abstract

Mediterranean phryganic ecosystems have been shaped for centuries by recurrent herbivory, yet the long-term ecological consequences of grazing cessation remain insufficiently resolved, particularly in protected island landscapes where conservation management often assumes that exclusion promotes recovery. In these drylands, the removal of grazing redirect assembly processes through shifts in dominance, heterogeneity, and functional strategy expression. Here, we use more than three decades-long grazing discontinuity within the Petrified Forest of Lesvos, an insular Mediterranean geopark, to examine how long-term herbivore exclusion reorganizes plant communities across taxonomic and functional dimensions. By integrating floristic inventories, multivariate community analysis, mixed-effects modeling, indicator species analysis, and community-weighted trait approaches, we reconstruct the ecological signature of grazing release in phryganic ecosystems. Long-term exclusion was associated with a broader species pool and a greater representation of protected taxa, while ungrazed communities exhibited lower Shannon and Simpson diversity, greater compositional dispersion, and a marked shift in dominance structure linked to the expansion of *Sarcopoterium spinosum*. Community differentiation was accompanied by directional reorganization of functional trait structure, with ungrazed plots characterized by taller vegetation and increased leaf and inflorescence length, indicating release from recurrent biomass removal and a transition toward more structurally expansive strategies. These results show that grazing exclusion does not simply enhance biodiversity, but reorganizes Mediterranean plant communities into an alternative ecological state shaped by altered competitive hierarchies, shrub-mediated filtering, and relaxed herbivory. In disturbance-structured island ecosystems, therefore, the ecological outcomes of protection depend not only on whether grazing is removed, but on how strongly community organization has historically depended on its continued presence.

## 1. Introduction

Mediterranean-type ecosystems are globally recognized as biodiversity hotspots, harboring exceptional levels of plant richness and endemism despite occupying a relatively small fraction of the global terrestrial surface [1,2]. Their vegetation structure and floristic composition reflect the combined influence of climatic seasonality and millennia of recurrent disturbances, particularly livestock grazing, wood harvesting, and fire, which have generated mosaics of semi-natural habitats maintained through dynamic equilibrium. Within these systems, disturbances have not merely degraded ecosystems but have also functioned as persistent ecological filters regulating competitive hierarchies [3,4], maintaining spatial heterogeneity [5], and enabling the coexistence of plant life forms adapted to seasonal drought and recurrent biomass removal [6,7]. However, when disturbance regimes are intensified, relaxed, or abruptly terminated, the resulting shifts in biotic interactions may reorganize community structure in ways that are not necessarily reversible or predictable [8,9].

Among the disturbance processes shaping Mediterranean dryland vegetation, grazing represents one of the dominant ecological processes structuring xeric Mediterranean phryganic ecosystems [10,11]. The ecological outcomes of grazing cannot be interpreted simply along a linear disturbance gradient; they emerge from complex interactions among disturbance regimes, plant functional strategies, and changes in land uses [12,13]. Through selective herbivory, livestock trampling, and nutrient redistribution [14], grazing modifies plant architecture, constrains vertical growth, shapes regeneration dynamics, and mediates dominance patterns within shrub–herb mosaics [15,16]. Variation in grazing intensity can therefore generate markedly different vegetation states [17,18], ranging from herbaceous-dominated communities under moderate grazing pressure to shrub-dominated systems where grazing is either excessive or absent [19]. In areas where grazing is absent or occurs at low intensity, plant communities tend to have more woody shrub species, while the presence of herbs decreases [20,21]. At moderate intensities, grazing may suppress competitively dominant growth forms and preserve fine-scale habitat heterogeneity. Under chronic overgrazing, however, vegetation can become structurally simplified, favoring species that can withstand heavy grazing pressure, such as certain annual herbs and grasses, and reducing the presence of woody shrubs and other less palatable species [22,23].

Such vegetation shifts are not merely compositional but are also expressed through changes in community-level functional trait distributions [24]. As species dominance patterns change, the distribution of ecological strategies within vegetation also shifts, a process that can be effectively captured through variation in plant functional traits [25]. Functional traits therefore provide a mechanistic framework for interpreting how grazing regimes shape community assembly [26,27]. In grazing-dominated ecosystems, plant persistence is often determined by morphological and functional traits related to plant architecture, growth strategy, and reproductive investment, enabling certain species to survive and even increase in abundance under repeated biomass removal [25]. Among these, plant height is consistently identified as one of the most grazing-sensitive traits of plant strategy, reflecting trade-offs between competitive light acquisition and exposure to herbivory [15,16]. Similarly, leaf size represents an important functional trait related to resource acquisition, water balance, and environmental tolerance, and has frequently been used to characterize plant responses to environmental gradients and disturbance regimes [28].

The ecological implications of these dynamics become particularly evident in systems where grazing regimes are intentionally modified. In protected areas, which are frequently managed under the implicit assumption that removing anthropogenic disturbance promotes biodiversity recovery and ecological restoration, grazing is often reduced or entirely excluded as part of conservation management strategies [17,29]. However, in ecosystems whose structure and diversity have long been shaped by herbivory, abrupt or prolonged grazing exclusion may relax disturbance-mediated filters that previously regulated plant interactions and vegetation structure. In semi-arid Mediterranean ecosystems, such relaxation frequently permits the expansion of competitively dominant growth forms, thereby redirecting successional trajectories and restructuring competitive hierarchies [15,30]; such shifts often manifest as shrub encroachment and increasing vertical stratification of vegetation [31,32], while they become detectable through changes in community-weighted mean trait values, which reflect the aggregate functional strategies represented within plant communities. These structural changes may compress niche space for subordinate taxa and alter regeneration dynamics, ultimately producing biodiversity outcomes that diverge from conservation expectations.

However, the ecological consequences of grazing within protected Mediterranean phryganic ecosystems remain comparatively understudied, with relatively few investigations explicitly examining its effects on plant communities [33], and even fewer within the Mediterranean Basin [34]. In these semi-arid shrublands, community responses to grazing may be strongly mediated by structurally dominant dwarf shrubs capable of persisting under both climatic stress and recurrent herbivory. Among these, *Sarcopoterium spinosum* (L.) Spach constitutes one of the most characteristic components of Mediterranean phrygana and degraded rangelands [35,36]. Its spiny morphology and drought tolerance enable persistence under intense grazing, while its canopy structure may influence neighboring vegetation through both competitive and facilitative interactions, a process widely documented for dominant shrubs in dryland ecosystems [37,38]. Under grazing pressure, *S. spinosum* may contribute to small-scale habitat heterogeneity by creating sheltered microsites for herbaceous plants. Yet when grazing intensity declines, expansion of shrub canopies may modify light availability and spatial occupancy, potentially restructuring competitive hierarchies and altering both species diversity and functional trait distributions within plant communities [31]. Understanding how grazing exclusion influences both species diversity and functional trait composition is therefore essential for clarifying how disturbance regimes shape community assembly processes in Mediterranean dryland ecosystems.

Lesvos Island, particularly its western region, provides a valuable natural comparative setting in which to investigate these dynamics. This region has experienced prolonged grazing pressure associated with traditional pastoral practices that have influenced vegetation composition and contributed to environmental degradation [36,39]. Within this landscape lies the Petrified Forest of Lesvos, an insular Mediterranean ecosystem dominated by phryganic vegetation, which is a protected natural monument incorporated within the Natura 2000 network. A fenced sector of the park has remained ungrazed for more than three decades, whereas adjacent areas continue to experience year-round sheep grazing under broadly comparable environmental conditions. Long-term grazing exclosures represent valuable natural experiments for assessing vegetation responses to disturbance release, as they allow the consequences of altered disturbance regimes to emerge through time [16,40]. The spatial contrast between grazed and ungrazed areas in the Petrified Forest Park therefore offers a rare opportunity to evaluate how sustained grazing exclusion influences plant diversity, vegetation structure, and community-level functional trait composition in Mediterranean phryganic ecosystems. Based on ecological theory regarding herbivory-mediated community assembly in Mediterranean shrublands, our aim was to evaluate whether long-term grazing exclusion corresponds with coordinated shifts in taxonomic composition, diversity structure, and functional trait distributions in Mediterranean phrygana. We hypothesized that: (i) plant community composition would differ between grazed and ungrazed sectors; (ii) grazing exclusion would modify local diversity patterns, potentially reducing Shannon and Simpson diversity through increased dominance despite permitting a larger overall species pool; (iii) variation in community structure and diversity would be associated with the abundance of the dominant shrub *Sarcopoterium spinosum*; and (iv) grazing exclusion would correspond with shifts in community-weighted trait values toward taller plants with larger vegetative and reproductive structures, consistent with reduced herbivory and relaxed disturbance filtering.

## 2. Methodology

### 2.1. Study Area

**Figure 1:**
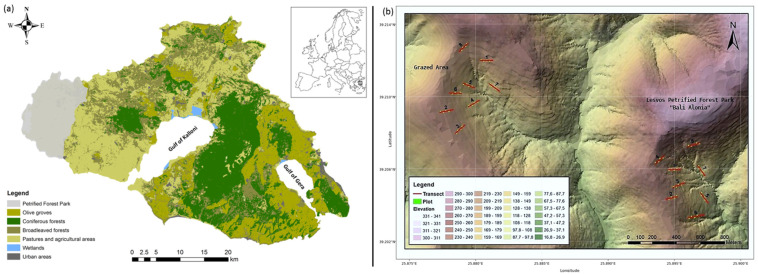
Geographic setting and detailed sampling layout of the study area in western Lesvos, Greece. (**a**) Land-use and vegetation cover of Lesvos Island, showing the Petrified Forest Park in western Lesvos and the geographic position of Lesvos within Europe. (**b**) Detailed topographic map of the study area showing the spatial arrangement of vegetation sampling transects in the grazed area and in the ungrazed sector within the fenced Petrified Forest Park (“Bali Alonia”). Red lines indicate the eight 100 m transects established in each grazing regime, while green squares represent the 10 × 10 m vegetation plots located along each transect. Numbers indicate transect identifiers used to distinguish the sampling units in the map. The background color gradient represents elevation (m a.s.l.), and longitude and latitude are provided for spatial reference.

The study was conducted in the western part of the Greek island of Lesvos, within the broader region of the Lesvos Petrified Forest Park (Figure 1a). The park forms part of the Natura 2000 network of protected areas (site: Lesvos—Western Peninsula—Petrified Forest; GR4110010 and GR4110003) and was designated as a protected natural monument in 1985 (Presidential Decree 443/85). The study site, locally known as Bali Alonia, lies within the protected zone of the park and contains exceptionally well-preserved fossilized tree trunks dating to the Early Miocene (~20 million years ago), representing ancestral taxa of modern Mediterranean flora and providing important evidence of the region’s paleoenvironmental history [41].

The ecological conditions of the region are shaped primarily by the semi-arid Mediterranean climate that characterizes western Lesvos. The climate is marked by hot, dry summers and mild, wetter winters, with mean annual precipitation of approximately 415 mm and a mean annual temperature of 17 °C, ranging from about 12.2 °C to 21.4 °C based on regional meteorological records from the University of the Aegean meteorological station.

Under these environmental constraints, vegetation in western Lesvos is dominated by xerophytic Mediterranean phrygana, a dwarf-shrub vegetation formation typical of semi-arid eastern Mediterranean landscapes. Phryganic vegetation is structurally dominated by the dwarf shrub *Sarcopoterium spinosum* (L.) Spach, a species widely recognized as a key structural component of Mediterranean drylands [35,39]. At the landscape scale, phrygana (pastures and agricultural areas) occupy approximately 76% of western Lesvos, while olive groves (*Olea europaea* L.) and oak formations (*Quercus* spp.) account for roughly 17%, and annual crops approximately 6.7% of the land surface [39,42,43].

The structure and persistence of these phryganic ecosystems have historically been strongly influenced by livestock grazing, which constitutes one of the dominant land-use practices across western Lesvos. Long-term pastoral activity has shaped vegetation composition, soil stability, and the spatial distribution of shrub and herbaceous communities [36,39], while minimizing the presence of alien species [44]. However, grazing pressure is not spatially uniform across the region. Within the Petrified Forest Park, livestock grazing has been excluded for more than three decades through the establishment of a metallic perimeter fence approximately 2.5 km in length, installed and maintained by the Lesvos Forest Department and the Natural History Museum of the Lesvos Petrified Forest. This long-term grazing exclusion has effectively created a large exclosure within an otherwise heavily grazed landscape, providing a valuable opportunity to evaluate vegetation responses to disturbance release under comparable environmental conditions.

### 2.2. Experimental Framework and Sampling Design

To evaluate the influence of grazing on plant community composition, diversity, and functional trait structure, we implemented a comparative field design contrasting two spatially adjacent grazing regimes. The two sites are separated by approximately 3 km and occur within the same geomorphological unit, thereby minimizing broad-scale environmental variation.

The first site was located within the fenced enclosure of the Petrified Forest Park, while the second site was situated outside the fenced park enclosure, where vegetation remains subject to continuous sheep grazing, reflecting the dominant pastoral land-use across western Lesvos. Site selection aimed to minimize environmental heterogeneity unrelated to grazing by restricting the comparison to areas with same geomorphological setting and broadly comparable elevation, slope exposure, geological substrate, surface rockiness, shading conditions, and vegetation physiognomy dominated by Mediterranean phrygana. Elevation values of plot centroids were broadly overlapping between the ungrazed and grazed sectors, ranging from 130 to 234 m a.s.l. inside the park and 157–225 m a.s.l. outside the park. Slopes also represented comparable hilly terrain, ranging from 5.6 to 25.6° inside the park and 3.8–16.8° outside the park. The grazed site experiences year-round grazing pressure, and in some locations low-intensity burning is applied as a localized rangeland management practice to reduce the cover of *S. spinosum* and promote herbaceous vegetation; however, such interventions are spatially restricted, do not represent a dominant disturbance regime at the landscape scale, and were avoided during sampling, as transects and plots were placed in continuously grazed sites outside visibly burned or recently burned patches. Within the Petrified Forest Park, by contrast, disturbance is limited to low-intensity visitor activity and localized vegetation removal associated with fossil site maintenance, resulting in substantially lower disturbance levels relative to the surrounding grazed landscape.

Vegetation sampling followed a hierarchical nested sampling design in order to capture plant community variation across multiple spatial scales. At each site, eight linear transects (100 m in length) were established across representative phryganic vegetation. The placement of transects within the park was strategically determined based on their proximity to established visitor pathways, while in the grazing area they started from paths that are either created by livestock activity or used by shepherds. Transects were then positioned within these comparable terrain conditions while avoiding abrupt topographic transitions, atypical microsites, areas affected by atypical disturbance or habitat discontinuity (Figure 1b).

Along each transect, four vegetation plots (10 m × 10 m) were established at regular 10 m intervals, yielding 32 plots per site. Plot spacing along transects ensured that sampling units captured independent vegetation patches while remaining within comparable environmental conditions. Within each plot, four randomly positioned subplots (0.5 m × 0.5 m) were sampled to record vegetation composition and plant functional traits. This nested sampling framework resulted in 128 subplots (0.25 m^2^) per site, providing spatial replication across transects and plots while allowing variation in species composition and functional traits to be quantified at fine spatial scales.

### 2.3. Vegetation Surveys

Field surveys were conducted during the main spring growing period of 2023 (March, April, and early May), when annual vegetation reaches peak development prior to summer senescence, while perennial and dwarf-shrub species are fully developed following winter growth, providing a suitable phenological window for reliable species-level identification and functional trait measurement. Within each 0.5 × 0.5 m subplot, all vascular plant taxa were recorded, and species richness was calculated as the total number of taxa present. All subplots were surveyed within the same seasonal window, thereby minimizing phenological bias between sites.

Plant taxa were identified primarily in situ. When field identification was not possible, specimens were collected and subsequently identified using standard regional floras and taxonomic references, including Flora Europaea, Flora of Turkey and the East Aegean Islands [45], La Flora d’Italia [46], Atlas of the Aegean Flora [47], and the Atlas of the Hellenic Flora [48]. For each subplot, the cover of every recorded species was estimated using the Braun–Blanquet cover-abundance scale [49]. All vegetation surveys were conducted by the same researcher (V.K.) to ensure consistency in species identification and cover estimation across sampling units.

### 2.4. Plant Functional Trait Measurements

Plant functional traits were measured for species contributing most strongly to vegetation structure within the study sites during the same sampling period as the vegetation surveys, ensuring consistency between trait measurements and recorded species composition. Trait measurements focused on the dominant species collectively accounting for approximately 80% of total vegetation cover across the sampled plots, following the dominant-species approach widely adopted in trait-based vegetation studies [50,51]. Functional traits were measured following standardized protocols for plant functional ecology [52]. For each selected taxon, measurements were taken from at least four individuals whenever sufficient individuals were available; when fewer individuals were present, all accessible individuals were measured.

Only fully developed, undamaged individuals were selected to minimize potential bias associated with ontogenetic stage or grazing damage. The following functional traits were measured in situ using a measuring tape: (a) maximum vegetative height (cm), measured from the ground surface to the highest photosynthetic tissue, excluding reproductive structures; (b) leaf length (cm), measured on fully expanded mature leaves; and (c) inflorescence length (cm) and, where appropriate, flower length (cm), measured on mature reproductive structures.

### 2.5. Ecological Attributes of Recorded Species

Ecological attributes were compiled for all recorded plant taxa following species identification. These attributes included life form according to the Raunkiær system as reported in the Atlas of the Hellenic Flora, chorological category, and endemism status, as well as the typical habitat types in which each species occurs. Information on species protection status under Greek legislation (Presidential Decree 67/81) and conservation status according to the Greek Red List of Threatened Species was obtained from authoritative national sources, including Dimopoulos et al. [53,54], the Atlas of the Hellenic Flora [48], and the National Environment and Climate Change Agency [55]. These ecological attributes were used to provide contextual information on the floristic composition of the sampled communities and to facilitate the ecological interpretation of vegetation patterns observed under contrasting grazing regimes.

### 2.6. Statistical Analyses

Species diversity at the plot level was quantified using the Shannon–Wiener diversity index (H′) and Simpson diversity index, both calculated from relative species cover values [56]. Shannon diversity was used to capture variation in richness and evenness, whereas Simpson diversity was included as a dominance-sensitive index giving greater weight to abundant species. Because vegetation composition was recorded using the Braun–Blanquet cover–abundance scale, ordinal cover classes were first transformed to quantitative percentage midpoints (r = 0.1, += 0.5, 1 = 2.5, 2 = 15, 3 = 37.5, 4 = 62.5, 5 = 87.5), following standard procedures in vegetation ecology for the numerical treatment of semi-quantitative relevé data [57]. These transformed values were then used to estimate relative species cover within each plot. Plots containing a single taxon had an H′ value of zero, reflecting complete dominance and the absence of evenness.

To investigate compositional responses of vegetation to long-term grazing exclusion, a species-by-plot community matrix was constructed from the transformed cover values. Species occurring in only a single plot were excluded prior to multivariate analysis in order to reduce the disproportionate influence of extremely rare taxa on ordination geometry and dissimilarity structure, a common procedure in community ordination analyses where such occurrences contribute minimally to overall compositional patterns but can introduce noise into distance-based analyses [58]. Because community matrices derived from Braun–Blanquet data are typically sparse and contain many zero values, compositional variation was analyzed using Bray–Curtis dissimilarity, which is well suited to semi-quantitative ecological community data. Bray–Curtis dissimilarity between plots *i* and *j* was calculated as

$${BC}_{ij}=\frac{\sum_{k=1}^{S}\left|x_{ik}-x_{jk}\right|}{\sum_{k=1}^{S}\left|x_{ik}+x_{jk}\right|}$$

where *x_ik_* and *x_jk_* are the transformed cover values of species *k* in plots *i* and *j*, respectively, and *S* is the total number of species included in the community matrix. Variation in plant community composition between grazed and ungrazed plots was first explored using non-metric multidimensional scaling (NMDS) based on Bray–Curtis dissimilarity, an approach well suited to sparse ecological community data and non-Euclidean species-response structure. Ordinations were computed in two dimensions using multiple random starts to ensure convergence on a stable solution, and ordination quality was evaluated using the stress statistic. Lower stress values were interpreted as indicating an adequate low-dimensional representation of compositional relationships among plots.

To test whether plant community composition differed between grazing regimes, permutational multivariate analysis of variance (PERMANOVA) was applied to the Bray–Curtis dissimilarity matrix [59], using grazing regime (grazed vs. ungrazed) as the principal explanatory factor. Because significant PERMANOVA results may arise either from differences in multivariate centroid location or from heterogeneity in within-group dispersion, homogeneity of multivariate dispersion was additionally assessed prior to ecological interpretation of the compositional differences. Environmental fitting (envfit) was then used to project continuous and categorical explanatory variables onto the NMDS ordination in order to evaluate whether the principal compositional gradients were associated with distance from paths and the cover of the dominant shrub *S. spinosum*.

To identify species exhibiting strong ecological association with either grazing condition, an indicator species analysis [60] was performed. This approach quantifies the degree to which species exhibit high fidelity (frequency within a group) and high specificity (restriction to that group), thereby identifying taxa characteristic of grazed versus ungrazed plots.

To evaluate whether grazing exclusion affected univariate diversity metrics while accounting for the hierarchical sampling design, a generalized linear mixed-effects model with a negative binomial error distribution was used for species richness (treated as count data), while a linear mixed-effects model was used for Shannon and Simpson diversity. In these models, grazing regime, distance from paths, and *S. spinosum* cover were treated as fixed effects in order to evaluate both the direct association of grazing regime with vegetation metrics and the potential contribution of the dominant shrub to variation in community structure, whereas transect identity was included as a random intercept to account for the non-independence of plots nested within transects.

Functional trait responses were then analyzed using community-weighted means (CWMs) calculated for each plot [24]. For each trait, species’ values were weighted by their relative cover in the corresponding plot, thereby capturing the dominant functional expression of the assemblage. Separate mixed-effects models were then fitted for plant height, leaf length, inflorescence length, and flower size using the same fixed- and random-effects structure described above. This allowed the evaluation of whether grazing exclusion was associated not only with shifts in taxonomic diversity and community composition but also with directional changes in community-level functional structure.

For all mixed-effects models, model adequacy was evaluated through inspection of residual distributions and fitted-versus-residual plots. Statistical significance of fixed effects was assessed using the appropriate inferential framework for mixed models, and all tests were interpreted at a significance threshold of α = 0.05. Model explanatory power was quantified using marginal and conditional pseudo-R^2^ values following Nakagawa & Schielzeth [61]. All analyses were performed in R [62] using functions implemented in packages appropriate for community ecology, mixed modeling, and indicator species analysis, including vegan, lme4, lmerTest, and indicspecies.

## 3. Results

### 3.1. Floristic Composition and Taxonomic Structure

**Figure A1:**
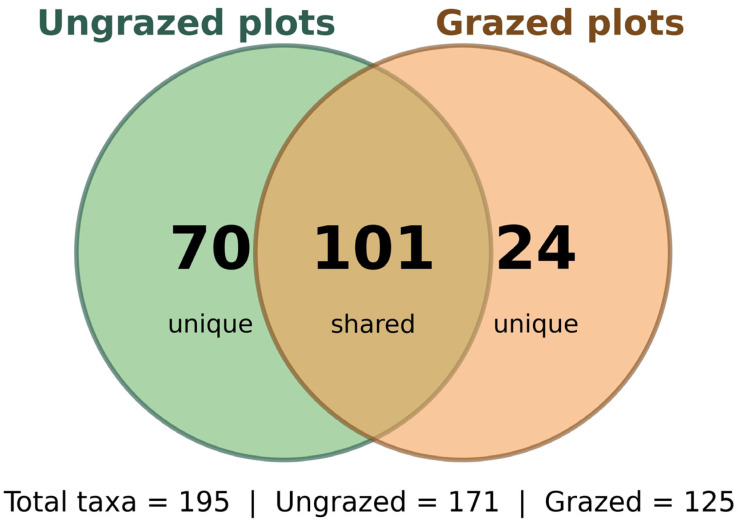
Venn diagram showing the number of vascular plant taxa recorded exclusively in ungrazed plots, exclusively in grazed plots, and shared between the two grazing regimes.

A total of 195 vascular plant taxa were recorded across the study area. Of these, 171 taxa belonging to 109 genera occurred within the Petrified Forest Park (ungrazed plots), whereas 125 taxa representing 84 genera were recorded in the adjacent grazed plots. The two sites shared 101 taxa, indicating a substantial overlap in species composition despite the contrasting grazing regimes. This overlap is further summarized in Figure A1, with 70 taxa recorded only in ungrazed plots, 24 taxa recorded only in grazed plots, and 101 taxa shared between both grazing regimes. All recorded taxa were native species, and none were identified as non-native or range-expanding within the study area. The floristic spectrum was dominated by a limited number of families, particularly Asteraceae, Poaceae, and Fabaceae, which together accounted for the largest proportion of taxa in both sites (Table 1).

The Raunkiær life-form spectrum was strongly dominated by therophytes in both management regimes (69.6% in ungrazed plots; 72.0% in grazed plots). Hemicryptophytes constituted the second most frequent life form (14.6% in ungrazed plots; 15.2% in grazed plots), followed by geophytes (9.9% in ungrazed plots; 8.8% in grazed plots) and chamaephytes (3.5% in ungrazed plots; 2.4% in grazed plots), whereas phanerophytes were nearly absent (0.6% in ungrazed plots; absent in grazed plots).

The chorological composition of the flora was similarly consistent between sites. Most taxa were associated with xeric Mediterranean phrygana and grassland habitats, and the majority belonged to the Mediterranean chorological category, reflecting the strong regional biogeographic signature of the vegetation in western Lesvos. In the ungrazed plots (Petrified Forest Park), Mediterranean taxa clearly prevailed, followed by East Mediterranean, European–Southwest Asian, and Mediterranean–Southwest Asian taxa. In contrast, the grazed plots showed a slightly different composition, where Mediterranean species remained dominant but were followed primarily by European–Southwest Asian and East Mediterranean elements, while Mediterranean–Atlantic taxa were comparatively more represented.

Despite this broad similarity in floristic structure, the ungrazed plots within the Petrified Forest Park supported a higher number of plant taxa, including a greater representation of species of conservation interest. Of the recorded taxa in the ungrazed plots, 12 out of 171 (7%) are protected under Greek legislation (Presidential Decree 67/81), including *Anacamptis collina*, *Anacamptis sancta*, *Ophrys argolica* subsp. *lesbis*, *Serapias bergonii*, *Serapias cordigera*, *Serapias orientalis*, *Serapias orientalis* subsp. *carica*, *Noaea mucronata*, *Ranunculus paludosus*, *Tulipa bithynica*, *Trifolium lappaceum*, and *Vicia cretica*, compared with only two species (*Anacamptis sancta*, *Ranunculus paludosus*; 1.6%) in the grazed plots. Among the taxa recorded within the park, *Tulipa bithynica* and *Trifolium mesogitanum* are classified as Vulnerable (VU), while the remaining species are categorized as Least Concern (121 taxa) or Not Evaluated (47 taxa). In contrast, within the grazed plots, 87 species are classified as Least Concern, 35 as Not Evaluated, and one species as Data Deficient.

### 3.2. Plant Community Composition

**Figure 2:**
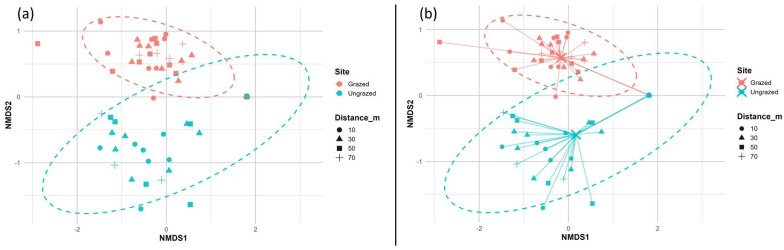
Non-metric multidimensional scaling (NMDS) ordination of plant community composition based on Bray–Curtis dissimilarity. (**a**) Ordination of vegetation plots from grazed and ungrazed sites, with ellipses representing 95% confidence intervals around group centroids. (**b**) Spider plot of the same ordination illustrating the dispersion of plots around the centroid of each grazing regime; lines connect individual plots to their respective group centroids, and line length reflects the degree of within-group compositional variability. In both panels, points represent vegetation plots, symbol shapes indicate distance from paths along transects (10, 30, 50, and 70 m), and the stress value of the NMDS solution was 0.147.

Multivariate ordination revealed pronounced differences in plant community composition between grazed and ungrazed plots. Non-metric multidimensional scaling (NMDS) based on Bray–Curtis dissimilarities yielded a stable two-dimensional solution (stress = 0.147), indicating an acceptable representation of the underlying multivariate relationships among vegetation plots. Plots from the grazed and ungrazed sites formed distinct though partially overlapping clusters in ordination space (Figure 2a). Vegetation plots within the grazed site were primarily concentrated in the upper portion of the NMDS configuration, whereas plots from the ungrazed site were primarily distributed along the lower portion of the ordination space. The spatial dispersion of plots revealed contrasting patterns of community variability between sites, even though a subset of taxa is shared between them. In addition to differences in centroid location, the ordination revealed contrasting patterns in the dispersion of plots around their group centroids. Vegetation plots from the grazed area formed a relatively compact cluster, suggesting a comparatively homogeneous plant community composition under continuous grazing. In contrast, plots within the ungrazed site showed a wider distribution across the NMDS space, indicating greater compositional heterogeneity within the protected area.

These differences in dispersion patterns are illustrated in the spider plot representation of the ordination (Figure 2b), where individual plots are connected to the centroid of their respective grazing regime. Distances between plots and centroids were generally shorter in the grazed site and longer in the ungrazed site, indicating that plant assemblages in grazed areas tend to converge toward a more uniform composition, whereas long-term grazing exclusion allows the emergence of more variable plant community structures. Symbols representing distance from paths along the sampling transects did not show any consistent directional arrangement across the ordination space, suggesting that distance from paths was not a major structuring factor of plant community composition within the sampled transects.

The patterns observed in the ordination were supported by permutational multivariate analysis of variance (PERMANOVA) based on Bray–Curtis dissimilarities using 999 permutations. When grazing regime was evaluated as the sole explanatory factor, plant community composition differed significantly between grazed and ungrazed plots (PERMANOVA: F = 10.62, R^2^ = 0.148, *p* = 0.001; 999 permutations; Table 2a). Grazing regime alone accounted for approximately 14.8% of the variation in species composition.

To further examine the drivers of community differentiation, a second PERMANOVA incorporated grazing regime, distance from paths, and the cover of the dominant shrub *S. spinosum*. Together, these predictors explained 31.7% of the total variation in plant community composition. Grazing regime remained the strongest associated factor influencing community structure (F = 12.37, R^2^ = 0.148, *p* = 0.001; 999 permutations), explaining the largest proportion of explained variance in the model. (Table 2b). Distance from paths also contributed significantly to compositional variation (F = 2.03, R^2^ = 0.073, *p* = 0.009), while the cover of *S. spinosum* accounted for an additional 9.6% of the variance (F = 7.98, R^2^ = 0.096, *p* = 0.001).

Because significant PERMANOVA results may arise either from differences in multivariate centroid location or from unequal dispersion among groups, the homogeneity of multivariate dispersion was subsequently evaluated using a permutation test for homogeneity of multivariate dispersion (betadisper). The analysis, consistent with the dispersion patterns of the NMDS spider plot (Figure 2b), revealed significant differences in dispersion between grazing regimes (F = 7.23, *p* = 0.009). Ungrazed plots exhibited larger distances to the group centroid, reflecting greater within-site compositional variability compared with the more compact clustering observed in grazed plots.

To further examine the environmental gradients associated with the observed compositional differentiation, explanatory variables were fitted onto the NMDS ordination using the envfit procedure. The cover of the dominant shrub *S. spinosum* showed a strong and highly significant association with the ordination configuration (NMDS1 = 0.9999, NMDS2 = 0.0110, r^2^ = 0.353, *p* = 0.001; 999 permutations), indicating that shrub dominance was closely aligned with the primary compositional gradient captured by the NMDS. Among the categorical predictors, grazing regime was also significantly associated with the ordination structure (r^2^ = 0.222, *p* = 0.001), reflecting the separation between grazed and ungrazed plots observed in the ordination space. The centroids of the two groups were positioned in opposite regions of the ordination (grazed: NMDS1 = −0.165, NMDS2 = 0.573; ungrazed: NMDS1 = 0.170, NMDS2 = −0.592), consistent with the compositional separation illustrated in Figure 2a. Distance from paths showed a weaker and marginally non-significant relationship with community composition (r^2^ = 0.091, *p* = 0.089). The centroids of the distance classes were distributed across the ordination space (10 m: NMDS1 = −0.373, NMDS2 = −0.070; 30 m: NMDS1 = −0.146, NMDS2 = −0.029; 50 m: NMDS1 = −0.101, NMDS2 = 0.056; 70 m: NMDS1 = 0.661, NMDS2 = 0.047), indicating limited structuring of vegetation composition along the sampled transects.

### 3.3. Species-Level Indicators of Grazing-Driven Community Differentiation

Species-level analyses showed that differences in community assemblages between grazing regimes were largely driven by a subset of statistically significant indicator taxa (Table 3a). In the ungrazed plots, the taxa with the highest indicator values included *Euphorbia peplus* (IndVal = 0.467, *p* = 0.001), *Avena barbata* (IndVal = 0.441, *p* = 0.001), and *Tragopogon campestre* (IndVal = 0.435, *p* = 0.001). Additional species significantly associated with ungrazed plots included *Lagoecia cuminoides*, *Asterolinon linum-stellatum*, *Moenchia mantica*, *Galium aparine*, *Vicia cretica*, *Anacamptis sancta*, and *Urospermum picroides*.

In contrast, several taxa were significantly associated with the grazed plots, where the highest indicator values were recorded for *Petrorhagia dubia* (IndVal = 0.673, *p* = 0.001), *Trifolium uniflorum* (IndVal = 0.567, *p* = 0.001), *Anthemis rigida* (IndVal = 0.566, *p* = 0.001), and *Trifolium stellatum* (IndVal = 0.503, *p* = 0.001). Additional species significantly associated with grazed plots included *Plantago coronopus*, *Trifolium campestre*, *Rumex bucephalophorus*, *Filago gallica*, *Plantago cretica*, and *Trifolium glomeratum*.

Indicator species analysis further revealed taxa associated with spatial position along the sampling transects. *Anagallis arvensis* and *Galium aparine* were significantly associated with the inner transect positions (10–30 m), whereas *Tyrimnus leucographus* was associated with the intermediate distance (30 m). The dominant shrub *S. spinosum* showed a significant association with the outer transect position (70 m), indicating a consistent spatial relationship with the most distant sampling plots (Table 3b).

### 3.4. Effects of Grazing Regime on Taxonomic Diversity

To assess whether grazing regime and local environmental gradients influenced plant taxonomic diversity, mixed-effects models were fitted for species richness, Shannon and Simpson diversity, while accounting for the hierarchical sampling structure of the study design.

Species richness was analyzed using a generalized linear mixed-effects model with a negative binomial error distribution. The model revealed a significant negative relationship between species richness and the cover of *S. spinosum* (β = −0.024 ± 0.005 SE, t = −4.380, *p* < 0.001; Table 4), indicating that increasing dominance of this shrub was associated with reduced numbers of plant species within plots. In contrast, neither grazing regime (β = −0.493 ± 0.313 SE, t = −1.576, *p* = 0.120) nor distance from paths (F_3,58_ = 0.171, *p* = 0.916) significantly affected species richness.

Shannon diversity, analyzed using a linear mixed-effects model, also declined significantly with increasing cover of *S. spinosum* (β = −0.027 ± 0.005 SE, t = −5.143, *p* < 0.001; Table 5a). In addition, Shannon diversity differed significantly between grazing regimes (F_1,58_ = 5.171, *p* = 0.027), with lower values observed in ungrazed plots compared with grazed ones (β = −0.588 ± 0.258 SE, *p* = 0.027). Distance from paths (F_3,58_ = 0.638, *p* = 0.594) did not significantly influence Shannon diversity.

The mixed-effects model explained a substantial proportion of the variation in Shannon diversity, with a marginal pseudo-*R*^2^ of 0.342 and a conditional pseudo-*R*^2^ of 0.564, indicating that fixed effects accounted for a considerable share of the observed variability while additional variance was captured by the hierarchical structure of the sampling design.

Simpson diversity, included as a dominance-sensitive complement to Shannon diversity, showed a comparable but more explicitly dominance-related pattern (Table 5b). Simpson differed significantly between grazing regimes (F_1,58_ = 5.117, *p* = 0.027), with lower values in ungrazed plots than in grazed plots. Simpson also declined significantly with increasing cover of *S. spinosum* (β = −0.0062 ± 0.0014 SE, t = −4.342, *p* < 0.001), indicating that dominance by this shrub was associated with reduced dominance-sensitive diversity. Distance from paths did not significantly affect Simpson diversity (F_3,58_ = 1.037, *p* = 0.383).

### 3.5. Functional Trait Responses to Grazing Regime

To determine whether grazing regime altered the functional structure of plant communities, community-weighted means of key plant traits were analyzed using linear mixed-effects models including grazing regime, distance from paths, and *S. spinosum* cover as explanatory variables. Across traits, grazing regime significantly influenced several community-weighted morphological traits, particularly those related to plant stature and reproductive architecture. In contrast, spatial position along paths did not significantly affect any of the functional traits examined (Table 6). Shrub dominance also contributed to trait variation, primarily affecting vegetative traits rather than reproductive characteristics.

Inflorescence length differed strongly between grazing regimes (F_1,58_ = 33.373, *p* < 0.001; Table 6(a)), with substantially greater values in ungrazed plots (β = 2.536 ± 0.439 SE, *p* < 0.001). Neither distance from paths (F_3,58_ = 0.621, *p* = 0.604) nor *S. spinosum* cover (F_1,58_ = 0.284, *p* = 0.596) significantly influenced this trait.

In contrast, flower size showed no detectable response to any of the predictors included in the model. Neither grazing regime (F_1,58_ = 1.046, *p* = 0.311), distance from paths (F_3,58_ = 0.719, *p* = 0.545), nor *S. spinosum* cover (F_1,58_ = 0.268, *p* = 0.607) significantly affected this trait (Table 6(b)).

Vegetative traits showed clearer responses to both grazing regime and shrub dominance. Leaf length differed significantly between grazing regimes (F_1,58_ = 7.353, *p* = 0.009; Table 6(c)), with longer leaves observed in ungrazed plots (β = 2.460 ± 0.907 SE, *p* = 0.009). At the same time, increasing cover of *S. spinosum* was associated with reduced leaf length (F_1,58_ = 4.558, *p* = 0.037; β = −0.039 ± 0.018 SE, *p* = 0.037). Distance from paths again showed no significant effect (F_3,58_ = 0.396, *p* = 0.756).

Plant height exhibited a similar response pattern (Table 6(d)). Vegetation in ungrazed plots was significantly taller than that in grazed plots (F_1,58_ = 18.478, *p* < 0.001; β = 24.652 ± 5.735 SE, *p* < 0.001), while plant height increased significantly with *S. spinosum* cover (F_1,58_ = 16.053, *p* < 0.001; β = 0.467 ± 0.116 SE, *p* < 0.001). As in the previous models, distance from paths did not significantly influence plant height (F_3,58_ = 0.170, *p* = 0.916).

Across functional trait models, marginal pseudo-*R*^2^ values ranged from 0.109 to 0.309, while conditional pseudo-*R*^2^ values ranged from 0.410 to 0.542, indicating that fixed environmental predictors explained a moderate proportion of trait variation, with additional variance attributable to hierarchical differences among sampling units.

## 4. Discussion

Our study provides a long-term, in situ evaluation of plant community reorganization associated with grazing exclusion in a Mediterranean dryland ecosystem, capitalizing on a naturally occurring, decades-long disturbance discontinuity within an insular Geopark. By jointly integrating taxonomic composition, local diversity, functional trait distributions, and multivariate community structure within an analytical framework, we capture multiple, interdependent dimensions of community assembly under contrasting disturbance regimes. In doing so, we move beyond conventional grazing studies that rely on short-term exclosures or single-response metrics [63,64], and instead we show how the sustained release from herbivory is associated with patterns emerging across hierarchical levels of ecological organization. These responses are expressed not only as shifts in species composition [34], but as changes in dominance structure and trait-mediated functional organization. Framed in this way, community responses to grazing exclusion can be interpreted as reflecting changes in assembly pathways operating across taxonomic and functional domains.

The significance of these findings lies not only in the patterns observed, but in the unique ecological context of our study system. The Petrified Forest of Lesvos functions as a de facto long-term ecological archive [65], preserving an unusually clear contrast in grazing history within a protected Mediterranean landscape, thereby creating the kind of long-duration ecological discontinuity that is rarely available for study in systems shaped by continuous human use. After all, in most Mediterranean drylands, disturbance regimes are spatially entangled, temporally unstable, and repeatedly modified by overlapping land-use practices [66,67], making it difficult to clearly disentangle how vegetation reorganizes when long-standing herbivory regimes are removed from the community assembly process, within otherwise comparable environmental conditions. Here, however, more than three decades of grazing exclusion provide access to the temporal domain in which community reassembly, dominance restructuring, and functional redistribution can be evaluated as patterns associated with a long-term disturbance discontinuity, rather than inferred as short-term responses.

At the same time, the structure of this natural comparison imposes an important interpretive constraint. Grazing regime and site identity cannot be fully disentangled, because the study contrasts one long-term ungrazed protected sector with one adjacent grazed sector. Although the sites were selected to be broadly comparable, unmeasured differences in soil depth, seed-bank composition, fine-scale microclimate, or historical land-use conditions prior to fencing may also have contributed to the observed patterns. Accordingly, our results should be interpreted as strong community-level associations with a long-term grazing discontinuity, rather than as definitive experimental isolation of grazing exclusion effects alone.

Within this framework, the floristic patterns observed indicate that long-term grazing exclusion did not generate a fundamentally different biogeographic assemblage, but rather modified the taxonomic expression of a shared Mediterranean flora. Both grazing regimes retained the same broad phytogeographical identity, with Mediterranean chorotypes clearly dominating, in agreement with established patterns in seasonally dry ecosystems where climatic filtering exerts a primary control over floristic composition [68]. Likewise, the predominance of therophytes, followed by hemicryptophytes, reflects a well-documented adaptive response to summer drought and environmental variability, whereby annual life cycles allow rapid exploitation of favorable seasonal windows [69,70]. At the same time, the ungrazed sector supported a substantially richer assemblage, both in terms of total taxa and generic representation, a pattern consistent with earlier observations from the same system during the initial years following grazing exclusion [71], suggesting that differences in species accumulation associated with disturbance release are not transient but persist over decadal timescales. Comparable levels of richness have been reported in other high-value protected landscapes in Greece such as the archeological sites at Peloponnesian Castles [72,73] and the Chelmos-Vouraikos National Park [74], further supporting the interpretation that long-term protection combined with reduced disturbance can enhance the floristic breadth of Mediterranean systems without altering their underlying biogeographic identity.

Beyond total richness, the higher representation of protected and conservation-relevant taxa within the ungrazed plots highlights a more subtle but ecologically important outcome of grazing exclusion. The exclusive occurrence of vulnerable species and the greater number of legally protected taxa within the Petrified Forest Park suggest that prolonged release from grazing may facilitate the persistence of species that are less tolerant to chronic herbivory or repeated biomass removal [75]. This pattern is consistent with studies showing that grazing can selectively exclude less tolerant or less competitive taxa, thereby constraining the conservation composition of plant communities [16]. From this perspective, grazing functions not only as a regulator of species abundance, but as a filter shaping which components of the regional flora are able to persist under long-term disturbance regimes [76,77]. Importantly, this implies that grazing regimes influence not only biodiversity quantity, but biodiversity identity, with direct consequences for conservation prioritization.

This selective role of grazing is further expressed at the scale of community organization. The multivariate analyses indicate that long-term grazing exclusion was associated with a clear restructuring of species assemblages, suggesting that grazing regime is strongly associated not only with patterns of species occurrence [16], but also with the structural organization of assemblages [78]. Importantly, this divergence emerged despite substantial floristic overlap, indicating that grazing exclusion reorganizes interactions within a shared species pool rather than generating a novel assemblage. In this sense, the compositional signal of exclusion reflects a reassembly of community organization rather than simple species replacement.

Equally important, the observed multivariate separation was accompanied by significant differences in within-group dispersion. Ungrazed plots occupied a broader area of ordination space and were significantly more dispersed around their centroid than grazed plots, indicating that long-term exclusion was associated not only with compositional divergence, but also with increased internal heterogeneity. This distinction should not be interpreted solely as a shift in centroid position (Figure 2), but as reflecting two complementary processes: a directional change in average community composition between grazing regimes and an expansion of compositional variability under exclusion. Ecologically, this suggests that continuous grazing tends to constrain plant assemblages toward a narrower and more homogeneous state [23], whereas its removal is consistent with the emergence of spatially differentiated assembly trajectories [30].

From this perspective, the comparatively compact clustering of grazed plots reflects the action of chronic herbivory. Recurrent grazing and trampling can simplify vegetation structure and favor taxa tolerant to biomass removal, promoting convergence toward a narrower range of compositional states [16,78]. By contrast, exclusion relaxes this constraint, enabling divergence in local assembly pathways and increasing compositional contingency [79]. This interpretation is consistent with the greater scatter of ungrazed plots in ordination space, indicating that the ecological consequences of grazing cessation are spatially uneven and internally differentiated, a pattern further supported by the emergence of distinct indicator species pools (Table 3) associated with each grazing regime.

In parallel, the strong association between *S. spinosum* cover and the primary ordination gradient further indicates that shrub dominance is central to this reorganization. The explanatory strength of *S. spinosum* in both PERMANOVA and envfit (Table 2) suggests that the shift in community composition under long-term exclusion is closely tied to the increasing structural influence of this dominant dwarf shrub. In Mediterranean phrygana, dominant shrubs can function as ecosystem engineers [80]; they alter light interception, space occupation, litter accumulation, and the spatial distribution of subordinate species [81,82]. The strong alignment of *S. spinosum* with the principal compositional axis therefore supports the view that grazing exclusion is consistent with a shrub-mediated pathway, in which the relaxation of herbivory enables the expansion of a structurally influential taxon that subsequently reorganizes the broader plant assemblage, with indicator taxa capturing this reorganization at the species level by reflecting the trait-mediated strategies favored under contrasting disturbance regimes [16]. This mechanistic interpretation is further reinforced by the negligible influence of within-transect spatial variation, as the contribution of distance from paths was comparatively minor, indicating that the primary compositional gradient was more strongly associated with grazing regime than with spatial position along transects.

The effects of grazing exclusion on taxonomic diversity reveal a clear decoupling between species richness and community diversity. Although the ungrazed sector supported a greater overall number of taxa, grazing regime did not significantly influence plot-scale species richness (Table 4), suggesting that the local species pool remains largely intact following the removal of herbivory. In contrast, Shannon and Simpson diversity declined under grazing exclusion (Table 5), indicating that shifts in relative abundance and dominance structure, rather than species presence alone, constitute the primary axis of diversity change. This demonstrates that grazing is linked to the regulation of diversity primarily through the control of dominance and evenness, rather than through species turnover per se. Under grazing, recurrent biomass removal appears to limit the development of strong dominance patterns, thereby maintaining more balanced species abundances and higher effective diversity. In contrast, the absence of grazing allows the redistribution of abundance toward a smaller subset of species, resulting in increased dominance and reduced evenness, despite the persistence of multiple taxa. Such decoupling between richness and dominance-sensitive diversity underscores that biodiversity responses to disturbance cannot be inferred from species counts alone, but require explicit consideration of abundance structure and community organization [83,84].

Importantly, the strong negative relationship between *S. spinosum* cover and both richness, Shannon and Simpson diversity indicates that these patterns are not directly driven by grazing regime itself, but by the structural consequences of its removal. In this context, diversity patterns emerge as a secondary outcome of changes in dominance structure, a mechanism consistent with theoretical and empirical work linking competitive dominance to reduced local diversity [8,82,85]. However, this dominance-driven restructuring is not only reflected in species abundances, but is underpinned by parallel shifts in the functional traits that define how species acquire resources and interact within the community.

The functional trait responses observed here provide a mechanistic extension of the compositional and diversity patterns discussed above, revealing how grazing regimes are associated with differences in community structure and dominant functional strategies expressed within plant assemblages. The consistent increase in plant height, leaf length, and inflorescence size under grazing exclusion indicates a shift toward structurally expansive growth forms, reflecting release from chronic biomass removal and greater expression of traits linked to vertical development, canopy expansion, and reproductive display. In grazed systems, recurrent herbivory acts as a strong selective filter that suppresses vertical development and favors low-stature, grazing-tolerant growth forms, whereas its removal permits the re-emergence of traits associated with competitive dominance and architectural expansion [16,25].

Among these traits, plant height represents a particularly informative axis of response, as it integrates the trade-off between light acquisition and herbivory risk and is consistently identified as one of the most grazing-sensitive plant characteristics across ecosystems [16]. The marked increase in height observed in ungrazed plots therefore reflects a shift in the primary selective pressure acting on the community, while the increase in leaf length suggests enhanced investment in photosynthetic surface area under reduced disturbance, consistent with patterns observed following herbivore exclusion in both Mediterranean and global dryland systems [86]. Moreover, the expansion of inflorescence size further indicates that reproductive structures are no longer constrained by repeated grazing, allowing greater allocation to reproductive display and potentially enhancing reproductive output under protected conditions [79]. In contrast, the absence of any detectable response in flower size suggests that not all reproductive traits are equally sensitive to grazing-mediated selection [40,87,88]. Unlike the other traits, which directly influence the likelihood of biomass removal, flower size may be more strongly constrained by phylogenetic, developmental, or pollination-related factors [89], and therefore less responsive to changes in herbivory pressure. This interpretation should nevertheless remain cautious, because flower size was necessarily evaluated only for taxa bearing suitable reproductive structures during the survey period; thus, the null result reflects the available flowering subset rather than a definitive absence of grazing-related sensitivity. Overall, this pattern suggests that grazing selectively filters those aspects of plant architecture most directly linked to herbivore access. At the trait level, the positive association between *S. spinosum* cover and plant height, together with its negative relationship with leaf length, indicates that shrub-dominated patches may favor vertical development while constraining lateral vegetative expansion, likely through light attenuation and spatial competition beneath shrub canopies [82,90]. Together, these compositional, diversity, and trait-level responses indicate that grazing exclusion is associated with coordinated changes across multiple dimensions of community structure. This functional interpretation should nevertheless be viewed within the seasonal scope of the sampling design. Because both grazing regimes were surveyed during the same spring peak-development period, phenological state was standardized across the comparison, including possible seasonal leaf variation in phryganic shrubs. The reported trait differences therefore describe the spring expression of community-weighted functional structure, rather than fixed year-round trait states, which would require repeated seasonal sampling to evaluate directly. Future research should combine replicated grazed–ungrazed comparisons across multiple Mediterranean drylands with repeated seasonal surveys and an expanded trait set, including SLA, LDMC, and belowground traits, to test whether the patterns identified here represent generalizable trajectories of post-grazing community reorganization.

Finally, our findings indicate that long-term grazing exclusion in Mediterranean drylands does not constitute a simple pathway to biodiversity recovery, but instead redirects community assembly toward alternative structural and functional states. Rather than increasing diversity in a unidirectional sense, the removal of herbivory is associated with a reorganization of plant communities through shifts in dominance, abundance distribution, and trait expression, producing outcomes that depend on which dimension of biodiversity is considered. In this context, grazing can be interpreted not merely as a disturbance to be removed, but as a regulating ecological process that stabilizes community structure by constraining dominance and limiting functional expansion. These results highlight the need for more nuanced management strategies in protected Mediterranean landscapes, where the decision to exclude or maintain grazing should be guided not by generalized assumptions, but by explicit ecological objectives regarding community composition, functional integrity, and conservation priorities.

## Figures and Tables

**Table 1:** Number of taxa per family recorded in the sampling plots inside the park and in the adjacent grazed site.

Family	Ungrazed Sites (Petrified Forest Park)	Grazed Sites
Apiaceae	6	3
Asparagaceae	1	-
Asteraceae	33	20
Boraginaceae	1	3
Brassicaceae	10	7
Caryophyllaceae	8	7
Chenopodiaceae	1	-
Cistaceae	2	4
Crassulaceae	4	1
Euphorbiaceae	2	3
Fabaceae	28	18
Fumariaceae	2	-
Geraniaceae	5	2
Hyacinthaceae	2	2
Iridaceae	2	2
Isoetaceae	1	1
Juncaceae	1	-
Lamiaceae	2	-
Liliaceae	2	2
Linaceae	2	1
Orchidaceae	7	1
Orobanchaceae	-	1
Papaveraceae	-	2
Plantaginaceae	-	4
Poaceae	29	28
Polygonaceae	3	2
Primulaceae	2	2
Ranunculaceae	2	2
Rosaceae	2	1
Rubiaceae	5	3
Scrophulariaceae	1	-
Valerianaceae	3	1
Veronicaceae	2	2

**Table 2:** Results of permutational multivariate analysis of variance (PERMANOVA) testing differences in plant community composition based on Bray–Curtis dissimilarity (999 permutations). Distance from paths was treated as a categorical factor with four levels (10, 30, 50, and 70 m).

Factor	df	R^2^	F	*p*-Value
*(a) Model testing the effect of grazing regime*				
Grazing regime	1	0.148	10.617	0.001
Residual	62	0.852		
Total	63	1.000		
*(b) Model including grazing regime, distance from paths, and S. spinosum cover*				
Grazing regime	1	0.148	12.367	0.001
Distance	3	0.073	2.027	0.009
*S. spinosum* cover	1	0.096	7.977	0.001
Residual	58	0.683		
Total	63	1.000		

**Table 3:** Indicator species associated with grazing regimes and distance from paths based on indicator species analysis. Significant associations were assessed using permutation tests (999 permutations).

**Species**	**Grazing Regime**	**Indicator Value**	***p*-Value**
*(a) Indicator species associated with grazing regimes*			
*Euphorbia peplus*	Ungrazed	0.467	0.001
*Avena barbata*	Ungrazed	0.441	0.001
*Tragopogon campestre*	Ungrazed	0.435	0.001
*Lagoecia cuminoides*	Ungrazed	0.387	0.002
*Asterolinon linum-stellatum*	Ungrazed	0.369	0.003
*Moenchia mantica*	Ungrazed	0.355	0.001
*Galium aparine*	Ungrazed	0.341	0.007
*Vicia cretica*	Ungrazed	0.333	0.001
*Anacamptis sancta*	Ungrazed	0.329	0.003
*Urospermum picroides*	Ungrazed	0.327	0.015
*Petrorhagia dubia*	Grazed	0.673	0.001
*Trifolium uniflorum*	Grazed	0.567	0.001
*Anthemis rigida*	Grazed	0.566	0.001
*Trifolium stellatum*	Grazed	0.503	0.001
*Plantago coronopus*	Grazed	0.492	0.001
*Trifolium campestre*	Grazed	0.491	0.001
*Rumex bucephalophorus*	Grazed	0.485	0.001
*Filago gallica*	Grazed	0.477	0.001
*Plantago cretica*	Grazed	0.475	0.001
*Trifolium glomeratum*	Grazed	0.466	0.001
**Species**	**Distance from paths**	**Indicator value**	***p*** **-value**
*(b) Indicator species associated with distance from paths*			
*Anagallis arvensis*	10–30 m	0.347	0.026
*Galium aparine*	10–30 m	0.330	0.047
*Sarcopoterium spinosum*	70 m	0.369	0.024
*Tyrimnus leucographus*	30 m	0.345	0.047

**Table 4:** Results of the generalized linear mixed-effects model evaluating the effects of grazing regime, distance from paths, and *Sarcopoterium spinosum* cover on species richness. The model was fitted with a negative binomial distribution and log link. Distance from paths was treated as a categorical factor (4 levels: 10, 30, 50, and 70 m; reference level: 70 m).

Predictor	Estimate (β)	SE	t	*p*-Value
Intercept	4.907	1.035	4.739	<0.001
Grazing regime	−0.493	0.313	−1.576	0.120
Distance 10 m	0.083	0.373	0.221	0.826
Distance 30 m	0.233	0.340	0.685	0.496
Distance 50 m	0.116	0.330	0.353	0.726
*S. spinosum* cover	−0.024	0.005	−4.380	<0.001

**Table 5:** Results of the linear mixed-effects model evaluating the effects of grazing regime, distance from paths, and *Sarcopoterium spinosum* cover on Shannon and Simpson diversity. Distance from paths was treated as a categorical factor (4 levels: 10, 30, 50, and 70 m; reference level: 70 m).

(**a**) **Shannon Diversity**				
**Predictor**	**Estimate (β)**	**SE**	**t**	***p*** **-Value**
Intercept	4.134	0.893	4.628	<0.001
Grazing regime	−0.588	0.258	−2.274	0.027
Distance 10 m	0.299	0.402	0.744	0.460
Distance 30 m	0.515	0.373	1.380	0.173
Distance 50 m	0.277	0.363	0.763	0.448
*S. spinosum* cover	−0.027	0.005	−5.143	<0.001
(**b**) **Simpson diversity**				
**Predictor**	**Estimate (β)**	**SE**	**t**	***p*** **-value**
Intercept	1.032	0.242	4.255	<0.001
Grazing regime	−0.158	0.070	−2.262	0.027
Distance 10 m	0.128	0.109	1.176	0.244
Distance 30 m	0.172	0.101	1.706	0.093
Distance 50 m	0.118	0.098	1.201	0.235
*S. spinosum* cover	−0.006	0.001	−4.342	<0.001

**Table 6:** Results of linear mixed-effects models evaluating the effects of grazing regime, distance from paths, and *Sarcopoterium spinosum* cover on community-weighted functional traits. Distance from paths was treated as a categorical factor (4 levels: 10, 30, 50, and 70 m; reference level: 70 m).

Trait	Predictor	Estimate (β)	SE	F/t	*p*-Value
(a) Inflorescence length	Grazing regime	2.536	0.439	33.373/5.777	<0.001
	Distance			0.621	0.604
	*S. spinosum* cover	0.005	0.009	0.284/0.533	0.596
(b) Flower size	Grazing regime	−3.084	3.016	1.046/−1.023	0.311
	Distance			0.719	0.545
	*S. spinosum* cover	0.032	0.061	0.268/0.518	0.607
(c) Leaf length	Grazing regime	2.46	0.907	7.353/2.712	0.009
	Distance			0.396	0.756
	*S. spinosum* cover	−0.039	0.018	4.558/−2.135	0.037
(d) Plant height	Grazing regime	24.652	5.735	18.478/4.299	<0.001
	Distance			0.170	0.916
	*S. spinosum* cover	0.467	0.116	16.053/4.007	<0.001

## Data Availability

The raw data supporting the conclusions of this article will be made available by the authors on request.
